# Harmonizing measurements: establishing a common metric via shared items across instruments

**DOI:** 10.1186/s12963-024-00351-z

**Published:** 2024-11-07

**Authors:** Iris Eekhout, Ann M. Weber, Stef van Buuren

**Affiliations:** 1 Department Child Health, Netherlands Organization for Applied Scientific Research TNO, Sylviusweg 71, Leiden, the Netherlands; 2 School of Public Health, University of Nevada Reno, Reno, NV USA; 3 University of Utrecht, Utrecht, The Netherlands

**Keywords:** Rasch model, Early childhood development, Global metric, Concurrent calibration, Meta-analyses

## Abstract

**Background:**

The proliferation of instruments that define instrument-specific metrics impedes progress in comparative assessment across populations. This paper explores a method to extract a common metric from related but different instruments and transform the original measurements into scores with a standard unit of measurement.

**Methods:**

Existing data from four assessment instruments of child development, collected from three different samples of children, were used to create “equate clusters” of items that measure the same behaviour in (slightly) different ways. A probability model was formulated to identify best items and groups to serve as anchors linking the instruments, assuming that items in an anchoring or “active” equate cluster are psychometrically equivalent. Quantification and inspection of item characteristic curves were used to resolve which equate clusters should be active. We simulated the impact of various analytic choices.

**Results:**

Simulation confirmed the feasibility of creating a common metric from data collected with different instruments from respondent samples with different abilities. The method performed as expected in an application in early childhood development.

**Conclusions:**

The use of equate clusters is an intuitive and flexible way to establish a common metric across instruments and facilitates the transformation of measurements obtained to a standardized scale. Standardizing instrument scores to a common metric allows for population-level comparisons on a global scale.

**Electronic supplementary material:**

The online version of this article 10.1186/s12963-024-00351-z) contains supplementary material, which is available to authorized users.

## Background

The proliferation of instruments that define instrument-specific metrics impedes progress in group and population-level comparative assessment. For example, there are over 150 instruments for measuring early childhood development (ECD) [5] that all aim to quantify ECD but differ in item selection, domain composition, reference values, age ranges, and languages. This wide variety is a mixed blessing. On the one hand, it is a hallmark of a healthy, thriving field of science since the tools cover many use cases of practical interest. On the other hand, each instrument defines its own set of scores, with no easy way to convert between them.

In contrast, many tools in the natural sciences produce measurements expressed in the International System of Units (SI). One may measure distance in many ways (e.g., by ruler, sonar, laser, or red-shift detection), and convert the results into the appropriate SI unit, the meter. A century of social and behavioural sciences has produced rather few standardised units. It thus remains challenging to aggregate data from multiple studies into a single data set, conduct integrative meta-analyses, compare populations, monitor change, evaluate treatments, or–in short–do cumulative science [3, 14]. Despite the difficulties of behavioural measurements [8], we need more work to standardise units to combat the field’s fragmentation and increase our ability to generate appropriate local and global priorities.

This paper focuses on deriving a scale unit *D* for ECD from existing data collected with different instruments. The D-score summarises the child’s development level with one number. In what follows, we assume the existence of a unidimensional latent scale on which we can position children (by ability) and items (by difficulty). The D-score quantifies the overall level of development and represents all domains (i.e., gross motor, fine motor, cognition, language, social-emotional, and communication). The intended use of the D-score covers the following use cases: to compare individuals, groups, and populations of children of the same age; to compare development within the same child, group, or population over time; to compare individuals, groups, and populations of different ages. To cater to these intended uses, the D-score must be an interval scale, i.e., a scale with a constant unit and an arbitrary zero.

We propose the Rasch model to provide the theoretical probability of a sequence of person responses to a set of measurement items (Rasch, 1960). The Rasch model is parsimonious and, critically, the estimated differences in difficulties between two items do not depend on the abilities in the calibration sample. This property is especially important in the analysis of combined data, where abilities can vary widely between sample populations. The D-score is expected to correlate highly with instrument-specific total scores that measure the same construct. The application of the D-score is restricted to measurement of children 0–3 years of age in this paper, but the D-score concept may well generalise to behavioural measurements at earlier (pregnancy) and later ages (childhood, puberty, adulthood).

Data harmonization with Rasch models have been applied in various ways in health-related assessments. For example, Prodinger et al. [20] outline a procedure where scores from different scales are harmonized by fitting the Rasch model to each scale and linking these through common items. Gross et al. [7] use an item banking approach where factor scores from confirmatory factor analyses are harmonized through common items across studies. Both procedures are iterative, so as the number of studies and instruments involved increases, the process can become cumbersome and complex. One alternative to this extensive data harmonization is concurrent calibration, which assumes that linked items across different instruments are identical in their measurement properties. Yet, this assumption is often difficult to validate, leading to potential inaccuracies. Moreover, respondents can only respond to one item within the linked item cluster.

In this paper, we explore a method to extract a common metric from related instruments and transform the original measurements into scores on that metric. Our four step approach, described in detail below, involves: (1) creating “equate clusters” of items that measure the same behaviour in (slightly) different ways, (2) formulating a probability model that restricts the difficulty estimates of items within an equate cluster to be identical; (3) evaluating the quality of equate clusters and identifying those that will serve as anchors linking the instruments in the final model; and (4) storing difficulty estimates from the final model in step 3 as a key, which provides the basis for estimating children’s ability with a common measurement unit, the D-score. We use a simulation study to investigate the method's performance under various analytic choices. We apply the method to derive D-scores from existing ECD data collected using four instruments across three countries, on three samples of children of varying ages. Comparisons across study and country groups can be made that would otherwise be precluded.

## Methods

### Data

We had access to item-level data from three studies in which four instruments were administered to measure ECD. The studies were included in a larger project to construct a generic score for child development that was performed by the Global Child Development Group (GCDG; [26, 31]). The Colombia study collected cross-sectional data by three instruments: 99 items from the Ages and Stages Questionnaire (ASQ; [24]), 84 items from the Denver Developmental Screening test (Denver; [6]), and 231 items from the Bayley Scales of Infant and Toddler Development, third edition (BSID-III; [1]), on 1311 children aged 0.5–3.5 years [23]. The Ethiopia study collected longitudinal data using 177 items from the BSID-III on 506 children aged 1–4 years [9]. The Netherlands study gathered longitudinal data for 55 items from the Dutch Development Instrument (DDI; [30]), on 2038 children aged 0–2.5 years [12]. Children received item sets appropriate for age. Table 1 provides an overview of the number of measurements by study and age group.

**Table 1:** Number of measurements by study and age range for three studies and four instruments

		Age range of respondents (years)					
Country	Study	0–1	1–2	2–3	> 3	N	Instruments
Colombia	GCDG-COL-LT42M	215	417	450	229	1311	BSID-III, Denver, ASQ
Ethiopia	GCDG-ETH	115	75	440	456	1086	BSID-III
Netherlands	GCDG-NLD-SMOCC	10,110	5120	1308	0	16,538	DDI
Total		10,440	5612	2198	685	18,935	

BSID-III = Bayley Scales of Infant and Toddler Development, third edition [1]; Denver = Denver Developmental Screening test [6]; ASQ = Ages and Stages Questionnaire [24]; DDI = Development Instrument [30]

### Bridging instruments and studies

There is no direct connection between the study from The Netherlands and the other two studies: the samples and instruments differ. Existing approaches to test equating, such as common-item non-equivalent groups design, non-equivalent anchor tests (NEAT) or vertical scaling [4, 11, 17], depend critically on the availability of common items. However, there may be no common items for instruments created independently, so classic approaches may fail, or work only under implausible assumptions.

This paper explores exploiting the overlap between instruments at the item level to build links between them. For example, many ECD instruments assess behaviours like: “child can sit without support”, “child says sentences of 2 words”, and so on, but they do so in different ways. If we could demonstrate that such variations have no or little impact on the measurement properties, then we could use such items to bridge instruments and studies by restricting their difficulty estimates to be identical.

**Fig. 1:**
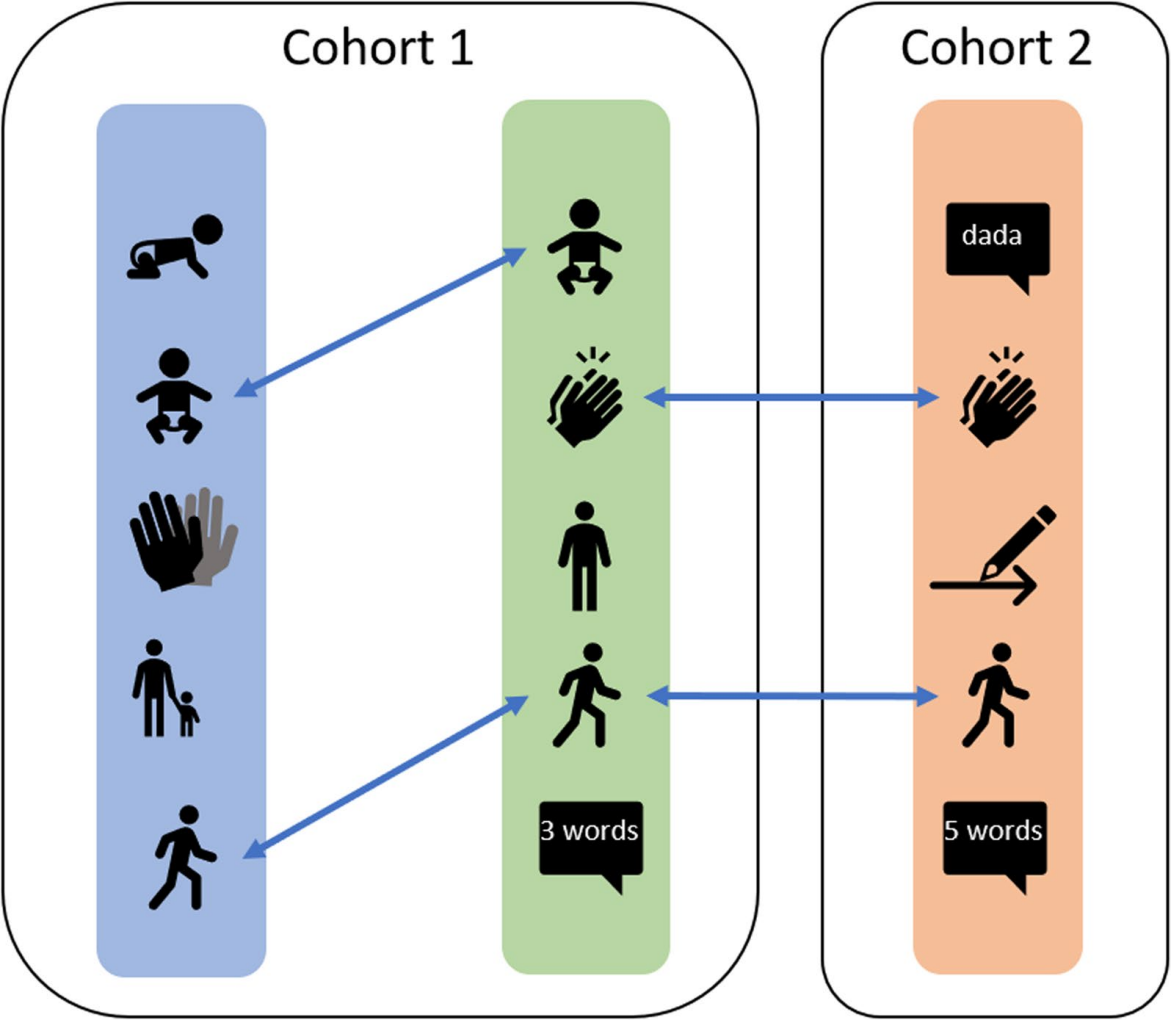
Example of three instruments linked by common items (i.e. equate clusters)

Let us look at an example. Figure 1 displays items from three fictitious instruments. The *blue* and *green* instruments were administered in *Cohort 1*. *Cohort 2* collected the data using the *orange* instrument. The instruments contain several common items, identified by the arrows between them. The item “walk alone” is common to all three instruments. The item "sits without support" is part of the *blue* and *green* instruments but not part of the *orange*. The item “claps hands” is part of the *green* and *orange* tools but does not appear in the *blue* instrument. When each cohort administers exactly one instrument, we can place common items in the same column of the data and estimate the model by vertical scaling. However, the situation is more complicated when a cohort collects data on two or more instruments. The *blue* and *green* instruments in Fig. 1 have two items in common. Column-wise stacking their responses would remove the information that the measurements pertain to the same child. Furthermore, the standard NEAT design does not handle the case where an item used in sample A matches to multiple items in sample B, for example, because sample B was administered multiple instruments (example not shown). In such situations, a more flexible way to link items is needed.

### Equate cluster method

The equate cluster method involves a four step modelling approach.

*Step 1*: Create equate clusters. We use the term “equate cluster” to refer to a group of items from different instruments that measure the same behaviour in (perhaps slightly) different ways. Since we do not know the set of common items in advance, as a first step, subject-matter experts identify potential bridges across studies by placing items into groups based on similarity.

*Step 2*: Formulate a probability model for equate clusters. The dichotomous Rasch model has two types of parameters (Rasch, 1960). Each item $i = 1, \ldots ,L$ has a difficulty parameter $\delta_{i}$, whereas each person $n = 1, \ldots ,N$ has an ability parameter $\beta_{n}$. The probability that person $n$ passes item $i$ depends on the difference $\beta_{n} - \delta_{i}$ through a logistic function:

$$\pi_{ni} = \frac{{{\text{exp}}\left( {\beta_{n} - \delta_{i} } \right)}}{{1 + {\text{exp}}\left( {\beta_{n} - \delta_{i} } \right)}}$$

The logarithm of the odds that a person with ability $\beta_{n}$ passes an item of difficulty $\delta_{i}$ is equal to the difference $\beta_{n} - \delta_{i}$. We opted for the dichotomous Rasch model for several reasons. The model is parsimonious, yields an interval scale, separates the ability and difficulty parameters, and is easy to adapt.

Based on an idea of Wright and Stone [33], we extended the Rasch model to support equate clusters. Wright and Stone fit separate Rasch models to each instrument and calculate the difficulty estimate for a hypothetically combined item by a weighted average of the separate difficulty estimates. We generalised their method to fit a constrained Rasch model that restricts the difficulty estimates of items within the same equate cluster to be identical. More formally, let $Q$ be the collection of items in equate cluster $q$. For an *active* equate cluster $q$, we restricted the difficulty parameters of the items in $Q$ as

$$\delta_{q} = \delta_{i} \,\,\forall i \in Q_{q}$$

where $\delta_{q}$ is the difficulty of the equate cluster. In practice, estimating the value of $\delta_{q}$ is done as follows. We first estimate the separate $\hat{\delta }_{i}$’s per item, combine them into $\hat{\delta }_{q}$ by their weighted average and replace each $\hat{\delta }_{i}$ by $\hat{\delta }_{q}$. Thus, if $w_{i}$ is the number of respondents for item $i$, the estimate for $\delta_{q}$ is

$$\hat{\delta }_{q} = \frac{{\mathop \sum \nolimits_{{i \in Q_{q} }} \hat{\delta }_{i} w_{i} }}{{\mathop \sum \nolimits_{{i \in Q_{q} }} w_{i} }}$$

The calculation is part of the iterative process for fitting the constrained Rasch model. Convergence is generally quick. Since we store items in separate columns, it is possible to test whether they can be equated without re-organizing the data. The approach provides elegant and flexible bridges for instruments with common items.

*Step 3*: Select the best items and identify active equate clusters (i.e. anchors). Since we do not know a priori whether items within an equate cluster are strictly equivalent, a formal modelling effort is required. The modelling task consists of selecting the items that best fit the Rasch model and identifying a set of homogenous equate clusters that span multiple instruments and are well-spaced along the latent continuum. Equate clusters are designated as active or inactive. An active equate cluster links items across instruments by restricting item difficulty estimates to be identical and thus are anchor items that bridge instruments. An inactive equate cluster does not enforce this restriction. High-quality equate clusters contain items that function similarly in different tests.

We evaluate the quality of an equate cluster by visual inspection of the item characteristic curves of each separate item in the cluster and comparisons of these curves to each other and to the fitted curve for the equate cluster. The distances between the item characteristic curves are evaluated as well as their slopes. These visual evaluations are supplemented with dedicated equate infit and outfit measures. *Equate infit* and *equate outfit* are generalized item fit statistics that measure the distance between the individual items in the group and the group item. Let ${z}_{ni}^{2}$ represent the standardised residual squared of person $n$ scoring item $i$ [32].We define equate outfit for equate cluster $q$ as the unweighted mean square over all responses on items that are members of $q$ as

$$u_{q} = \mathop \sum \limits_{{i \in Q_{q} }} \mathop \sum \limits_{n}^{{w_{i} }} z_{ni}^{2} /w_{q} w_{i} ,$$

where $w_{q}$ is the number of items in equate cluster $q$. If $n\left( q \right) = 1$ then we obtain the conventional item outfit statistic. Likewise, we define *equate infit* as

$$\nu_{q} = \mathop \sum \limits_{{i \in Q_{q} }} \mathop \sum \limits_{n}^{{w_{i} }} W_{ni} z_{ni}^{2} /w_{q} \mathop \sum \limits_{n}^{{w_{i} }} W_{ni} ,$$

with $W_{ni}$ the variance of the observed response of person $n$ to item $i$ as defined in Wright & Masters [32] (p. 100). Since these quantities are also $\chi^{2}$-statistics, we may interpret them like item infit and outfit.

We use infit and outfit to quantify how well persons, items and equate clusters fit the Rasch model. The outfit statistic is the $\chi^{2}$ statistic of the residual and is sensitive to model deviations in the tails. The infit statistic weighs down the extremes and is informative about the fit near the middle of the distribution. Person and item fit are considered satisfactory if the values stay below 1.5. For equate infit, there are no conventions, and we propose the use the same cut-off of 1.5. Since the equate clusters are instrumental in the model, it is useful to assess items within an equate cluster for differential item functioning (DIF). DIF tests are typically used to evaluate the similarity of item functioning across different groups of respondents. The current application emphasizes between instruments. For the Rasch model, a DIF test can be performed using an ANOVA to evaluate differences in standardized residuals between items within an equate cluster at each ability level [25]. The model is defined as:

$$z_{ni} = \beta_{n} + G_{i} + \beta_{n} *G_{i} + \in_{ni}$$

where $z_{ni}$ represents the standardized residual for person $n$ on item $i$ in the restricted model, $G_{i}$ is the effect of the *i-*th item, and $\in_{ni}$ is the random error term associated with the *n*-th observation in the *i*-th item, assumed to be normally distributed. A significant F-test for the inter-person-group variance indicates DIF. The magnitude of uniform DIF can be quantified using eta squared ($\eta^{2}$), which represents the proportion of total variance attributed to the item effect. Severe DIF may warrant deactivation of the equate cluster, and the following thresholds are recommended to interpret the severity of DIF based on $\eta^{2}$: $\eta^{2}$ = 0.01 for a small effect, $\eta^{2}$ = 0.06 for a medium effect, and $\eta^{2}$ = 0.14 or greater indicates a large effect [2].

Step 4: Store the key and estimate ability. The set of difficulty estimates from the final model of step 3 is stored as a key (i.e. item parameter calibration). The key freezes the measurement and provides the basis for quantifying person abilities. Estimated abilities from the same key can be compared, even if the basic measurements were made by different instruments.

To estimate the constrained Rasch model, software was developed in an R package called dmetric [27]. This package contains various tools to work with equate clusters (see Additional file 1). The ‘rasch()’ function in dmetric package extends the ‘rasch.pairwise.itemcluster()’ function from the sirt package [21, 22]. The dmetric package also includes functions that calculate infit and outfit and visualise item response curves. At the time of writing, dmetric is not yet available on CRAN. Please contact the package authors for access.

## Simulation

### Objective

Several studies provided insight into the performance of common-item equating methodology [10, 15, 16, 18]. What is still missing is an evaluation of the quality of the constrained solution using equate clusters as proposed above. The simulation design provides answers to the following open questions:

- What is the optimal number of equate clusters and their location along the scale continuum?
- What is the impact of disparate ability distributions obtained from different samples?
- How does the method perform under equate cluster misspecification (i.e., items differ in difficulty)?

### Simulation design

Table 2 presents the parameters of the simulation design. Item response data were simulated for either two or three instruments. Each instrument contained 10 unique items plus one, two or five additional items in equate clusters. Item difficulties had different amounts of overlap between instruments: Item difficulties (1) did not overlap and were not close: [− 5,− 3] and [3,5]; (2) did not overlap but were close: [− 3,− 0.1] and [0.1,3]; or (3) overlapped: [− 2,1] and [− 1,2]. In the base scenario, the difficulties of items in equate clusters were set equal in all instruments. We simulated "wrong" equate clusters by gradually increasing this difference across instruments, starting from 0 (no deviation) to 2 logits in steps of 0.1 logits.

**Table 2:** Summary of the conditions in the simulation design

Parameter	Variation	Number of variations
Number of instruments^a^	2 or 3	2
Difficulty ranges for the items in the instruments $\left( {\delta_{i..l} } \right)$	No overlap: [− 5,− 3] and [3,5] Close: [− 3,− 0.1] and [0.1,3] Overlap: [− 2,1] and [− 1,2]	3
Number equate clusters	1, 2, or 5	3
Location equate clusters	In the centre of the instruments In range of one instrument (not the other) Evenly spread over both instruments At the extreme end of the instruments	4
Equate misspecification	Difficulty deviation of 0 to 2 logits with steps of 0.1	21
Abilities ($\beta_{n}$)^b^	Equal: N(0 ~ 1) Different: N(− 1, 1) and N(1, 1) (2 instruments) or N(− 1.5, 1),N(0.5, 1), and N(2.5, 1) (3 instruments)	2

^a^Each instrument contained 10 items, with additional equate items

^b^Data were generated for 1000 persons per instrument

We hypothesised that the best locations for equate clusters would be relatively far apart and cover a wide scale range. Equate clusters were placed in the centre of the instruments, in the full range of one instrument but not in the other, spread equally over both or in the extreme of one instrument. In the base scenario, person abilities in both samples had the same normal distribution N(0, 1). We increased the difference between means to 2, so N(− 1, 1) and N(1, 1), and simulated sample distributions for three instruments as N(− 1.5, 1), N(0.5, 1) and N(2.5, 1).

The ability and item parameters settings were input to the ‘sim.raschtype()’ function of the sirt package to generate the data (see R code in Additional file 1) [21]. We fitted a Rasch model on the full data to obtain the true difficulty parameters for the reference situation where all items were administered to all respondents. Subsequently, the data were split such that 1000 persons had data for the first instrument, another 1000 for the second and, if the condition required, another 1000 for the third instrument. Two additional Rasch models were fitted to these data: one where the equate cluster items had the same difficulty and another where all item parameters were estimated freely (i.e., thus, without any active equate clusters). We compared the estimated difficulties from these two Rasch models to the reference values from the full data.

### Model performance

We calculated the correlation (ρ) between the true and estimated difficulty, with and without active equate clusters. A higher correlation indicates a closer approximation of the true difficulties.

**Fig. 2:**
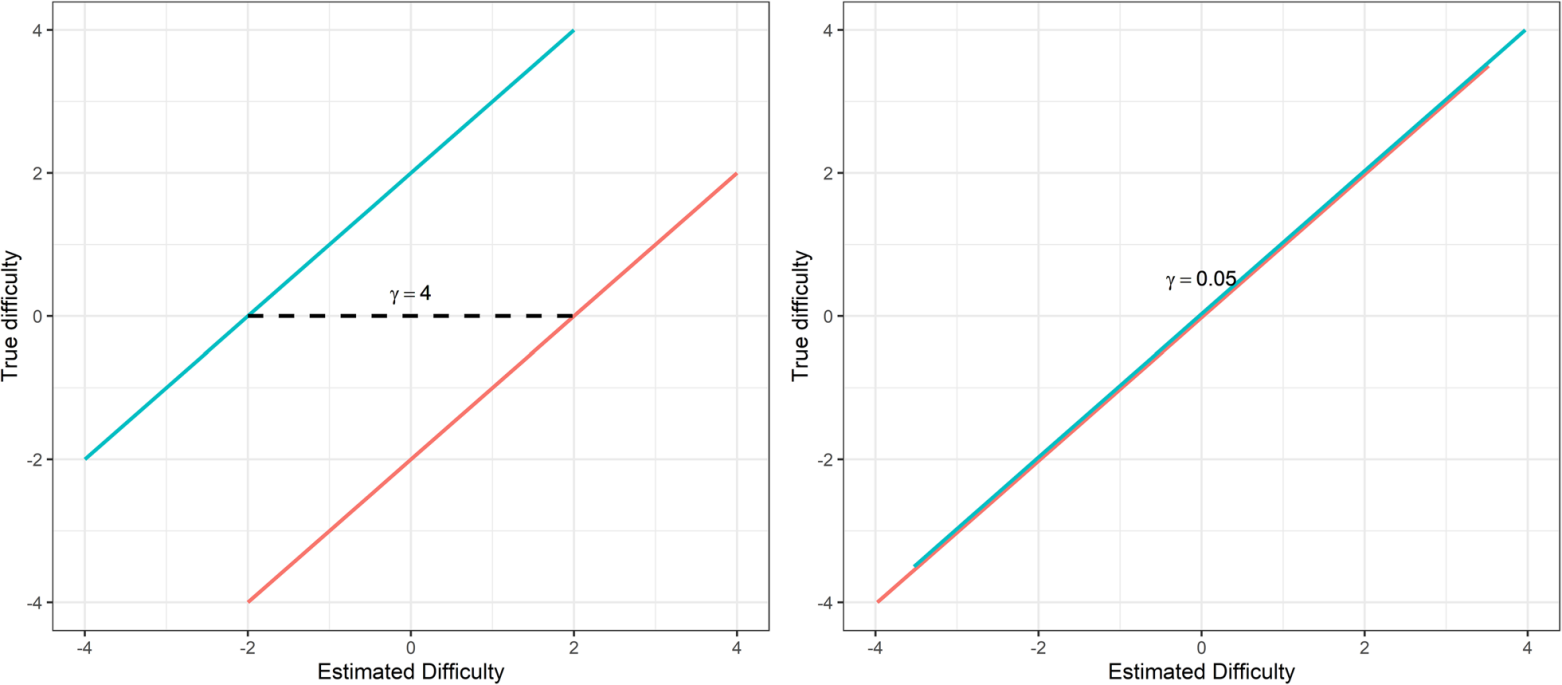
Illustration of the mis-alignment parameter, the left panel illustrates large mis-alignment between two instruments ($\gamma$ = 4) and the right panel a small misalignment ($\gamma$ = 0.05)

Misalignment ($\gamma$) was measured by the vertical distance between two lines (Fig. 2), one for instrument A and one for instrument B. The coefficient for misalignment

$$\delta = c + b\hat{\delta } + \gamma k$$

captures how well the estimated difficulty parameters reproduce to the same scale. Here *δ* is the true difficulty parameters of the items, c is the constant, *b *is the coefficient for $\hat{\delta }$ which are the estimated difficulties, and $\gamma$ is the misalignment for instruments *k*. Lower values are better.

## Simulation results

### Correct equate cluster specification.

**Fig. 3:**
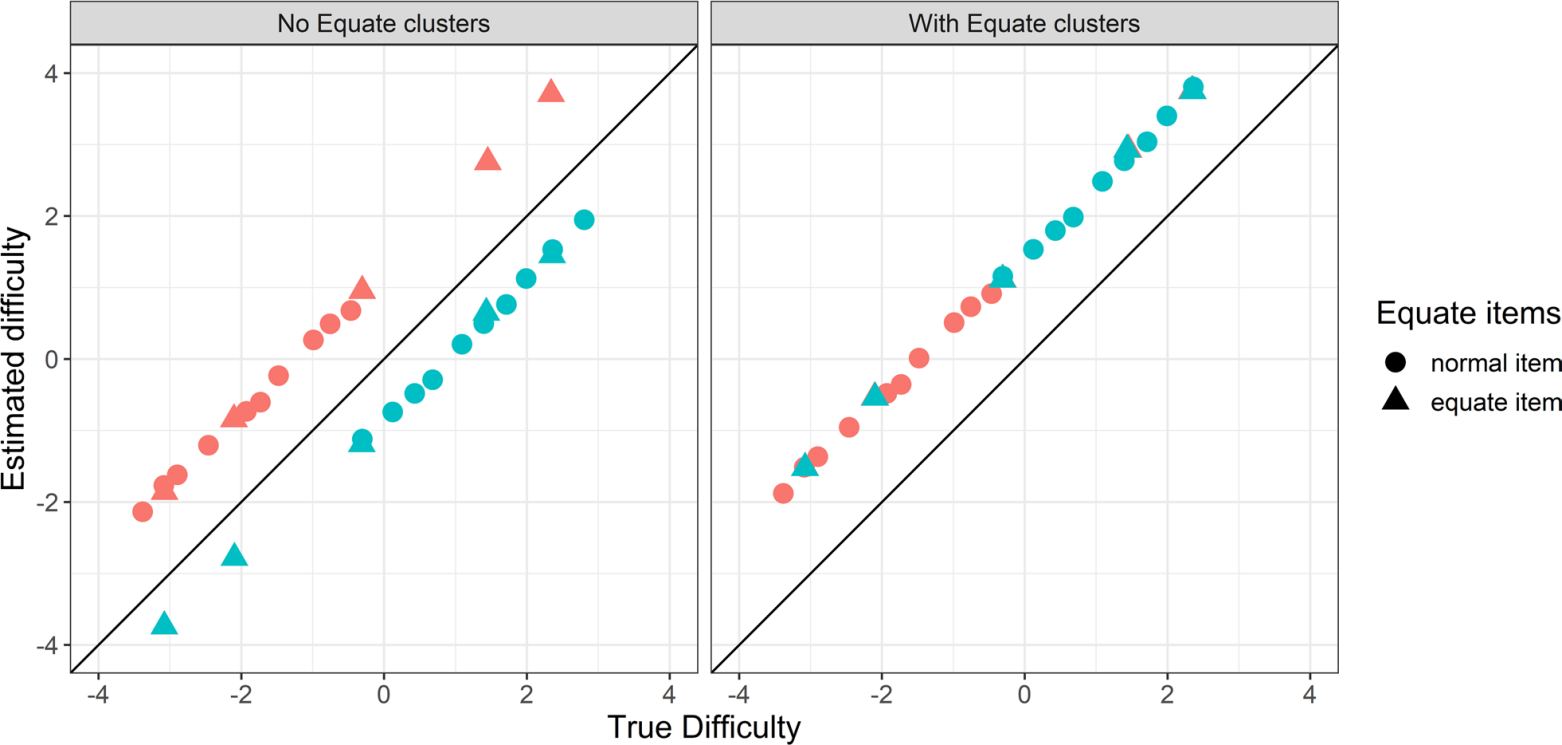
Difficulty estimates from model without active equate clusters (ρ = 0.85; γ = 1.94) (left) and model with active equate clusters (ρ = 0.99; γ = -0.03) (right) where difficulty ranges are close, cohort abilities differ and five equate clusters are spread through both instruments. Difficulty estimates are coloured by instrument

Table 3 summarises the most salient findings. When the equate clusters are correctly specified, correlations between the estimated and true difficulties exceed 0.99, and misalignment is small (γ < − 0.3 logits). The model without active equate clusters works well only if ability distributions are equal and difficulties are overlapping. In other cases, the misalignment is substantial, or the correlation is low. Equate clusters positioned in the tail of the ability distribution are slightly less successful in recovering the true parameters. The number of equate clusters had little effect. The left-hand side panel in Fig. 3 presents an example of severe misalignment (γ = 1.94) when sample abilities differ for the model without active equate clusters. The model with equate clusters yields difficulty estimates with near-perfect alignment (γ = − 0.03) and high correlation with the true values (ρ = 0.99). Additional file 2 provides a full tabulation of the results.

**Table 3:** Simulation study results to compare the model with equate clusters to the model without equate clusters

		*No Equate clusters*		*With Equate clusters*	
*Difficulties*	*Abilities*	*ρ*	*γ*	*ρ*	*γ*
No overlap	Equal	0.997	0.625	0.999	0.161
Close	Equal	0.996	0.394	0.999	− 0.010
Overlap	Equal	0.996	0.132	0.998	0.032
No overlap	Different	0.963	2.460	0.997	0.243
Close	Different	0.832	2.090	0.999	0.000
Overlap	Different	0.779	1.810	0.998	− 0.008

ρ is the correlation between the estimated and the true difficulties, γ is the mis-alignment. The results are averaged over the other conditions

### Equate cluster misspecification

An equate cluster is incorrectly specified if its items differ in difficulty. Large misspecification affects the model's performance. Sometimes, using something other than equate clusters may be better. Additional file 3 shows the amount of misspecification (in logits) needed to make the model without equate clusters better than the model with equate clusters. When the sample abilities are different, the model with active equate clusters wins unless the amount of misspecification is dramatically large (say > 2.0 logits). When the sample abilities are similar, equating is less tolerant to misspecification. For example, when the instruments are sensitive to different ranges of the latent scale, the amount of misspecification should be lower than 0.5 logits. For strictly parallel measures with the same working range, equating is superior only if the amount of misspecification is lower than 0.2 logits.

## Application to existing data

We fitted the Rasch model on the combined data in Table 1, with and without active equate clusters. We applied a strict item selection based on the fit of the items to the Rasch model to select only the best items for the combined scale. Accordingly, items were removed based on item infit and outfit (< 1.5) until the model contained only items with excellent fit. The fitted model included 185 remaining items: 31 ASQ items, 84 BSID-III items, 53 DDI items, and 17 Denver items. Eleven candidate equate clusters were carefully selected based on expert judgement. Also, we calculated infit and outfit statistics for active equate clusters. The resulting solution connects the studies by eight active equate clusters. Table 4 provides an overview of items per study and how these are connected.

**Table 4:** Overview of the equate clusters used to link the three studies and four instruments

	ASQ 31 items	BSID-III 84 items	DDI 46 items	Denver 17 items
Netherlands N = 2038 Rows = 16,650			EQ1; EQ2; EQ3; EQ5; EQ6; EQ8	
Ethiopia N = 506 Rows = 1089		EQ1; EQ3; EQ4; EQ5; EQ6; EQ7; EQ8		
Colombia N = 1311 Rows = 1311	EQ4; EQ7	EQ1; EQ3; EQ4; EQ5; EQ6; EQ7; EQ8		EQ1; EQ2; EQ1; EQ8

N = the number of children in the study; Rows = the number of measurements; BSID-II = Bayley Scales of Infant and Toddler Development, third edition [1]; Denver = Denver Developmental Screening test [6]; ASQ = Ages and Stages Questionnaire [24]; DDI = Development Instrument [30]

**Fig. 4:**
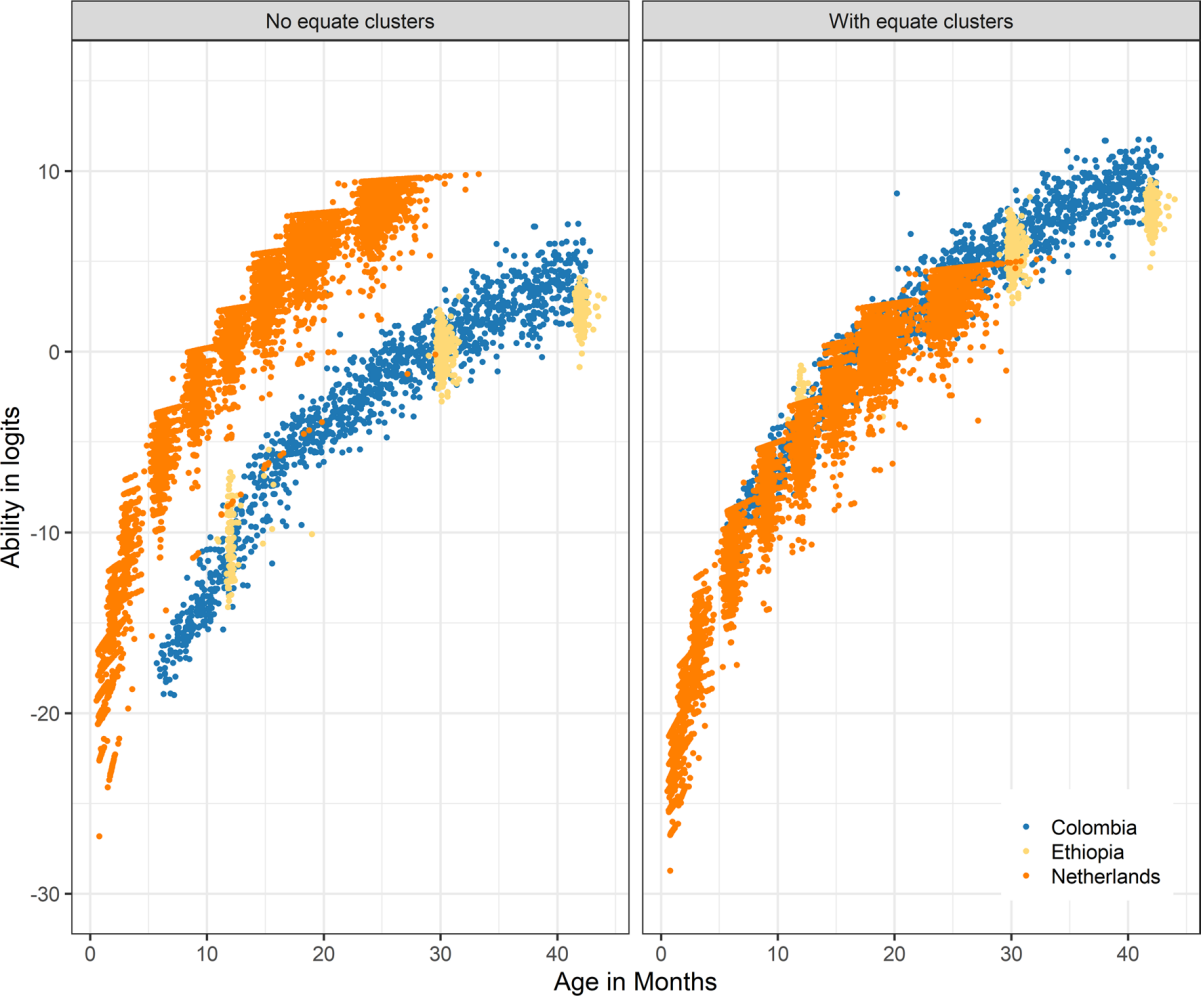
Latent ability in logits for age for the three studies. The left panel results from the model without equate clusters and the right panel results from the model with equate clusters

**Fig. 5:**
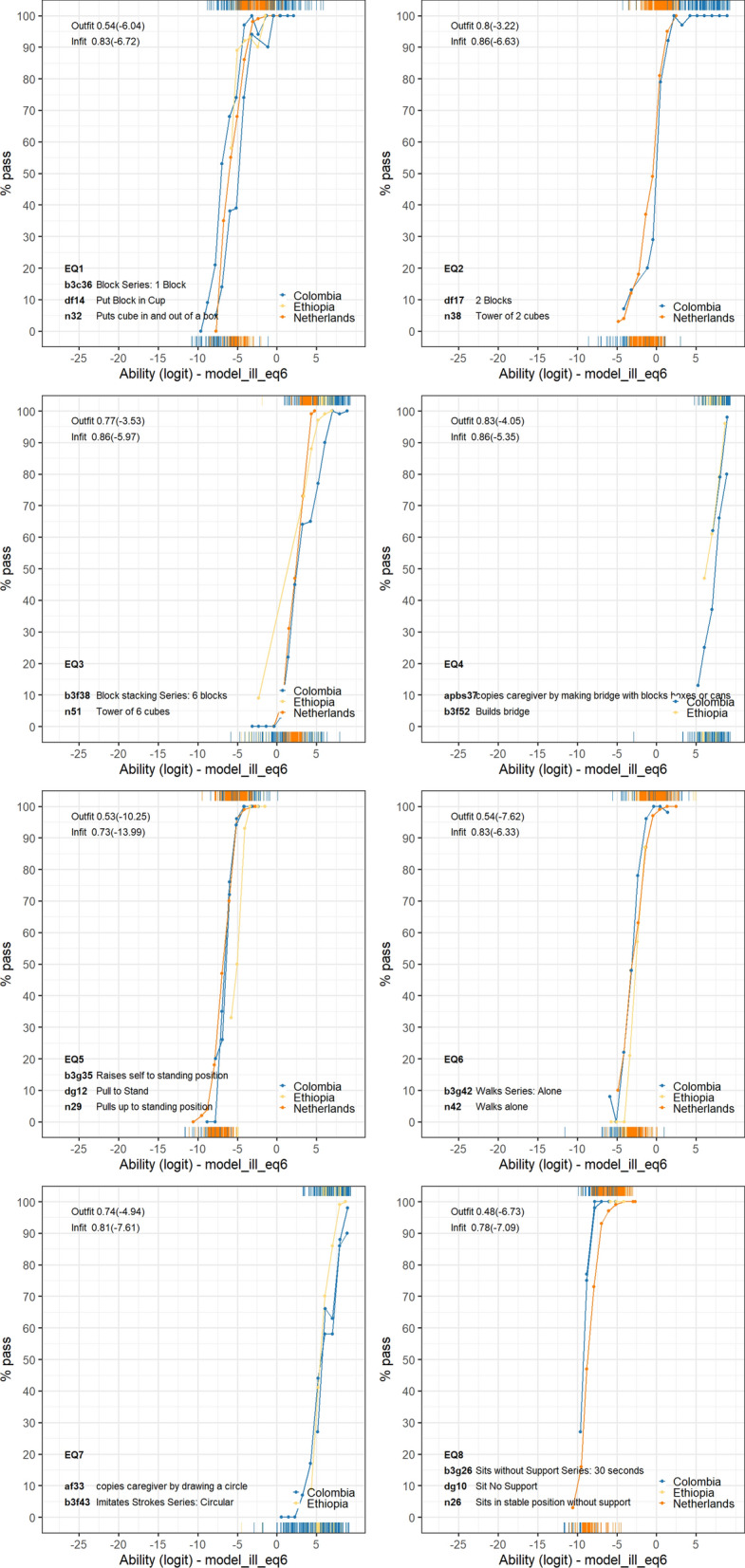
Percentage pass for ability in the data for the equate clusters

Figure 4 displays the latent ability scores for the model without (left panel) and with (right panel) equate clusters. The model without equate clusters places the ability distribution per study around the global mean, thus severely distorting the association with child age. This solution is not suitable for comparing child development across studies. The model with equate clusters resolves these issues and results in one common scale. One might wonder why Ethiopia and Colombia appear on a similar scale, even without equate clusters. The reason is that these studies have common BSID-III items. As we may expect, the estimated item difficulties differ between the models with and without equate clusters. Table 5 presents the difficulty parameter estimates for items in equate clusters, for each model. For example, when we place items "Sit no support" from Denver, "Sits without support (30 s)" from BSID-III and "Sit in stable position without support" from the DDI into an equate cluster, these have a common difficulty of − 8.815. In the unconstrained model, item difficulties vary widely (− 17.935, − 18.213, − 2.184, respectively), which destroys the common scale. The DIF analysis within the item clusters revealed significant F-tests for EQ1, EQ3, EQ5 and EQ6. However, the differences in logits between the difficulty estimates of individual items and those of the equate cluster were less than 0.2 logits. The simulation study showed that with a mis-specification smaller than 0.2 logits, using an equate cluster outperforms not using an equate cluster. Additionally, the effect sizes were negligible, with all ${\eta }^{2}$ values being less than 0.01 (Table 5). Figure 5 displays the probability to pass the item by ability for each equate cluster. These plots confirm that differences between the item characteristic curves from the different studies and instruments are small, as desired.

**Table 5:** Overview of the difficulty estimates for the items in equate clusters with DIF test results

					Model without equate clusters		Model with equate clusters			
Equate	Item	Instrument	Label	N	${\delta }_{i}$		${\delta }_{q}$	${\delta }_{i}$	${\delta }_{q}-{\delta }_{i}$	${\eta }^{2}$
EQ1	b3c36	Bayley3	Block Series: 1 Block	555	− 14.042		− 5.703	− 5.841	0.138	0.0025*
EQ1	df14	Denver	Put Block in Cup	254	− 12.462		− 5.703	− 5.612	− 0.091	
EQ1	n32	DDI	Puts cube in and out of a box	3171	0.654		− 5.703	− 5.688	− 0.015	
EQ2	df17	Denver	2 Blocks	514	− 6.882		− 0.371	− 0.386	0.015	0.0011
EQ2	n38	DDI	Tower of 2 cubes	2896	5.659		− 0.371	− 0.369	− 0.002	
EQ3	b3f38	Bayley3	Block stacking Series: 6 blocks	1441	− 2.529		3.017	3.058	− 0.041	0.0019*
EQ3	n51	DDI	Tower of 6 cubes	1573	8.642		3.017	2.978	0.039	
EQ4	apbs37	ASQ-I	copies caregiver by making bridge with blocks boxes or cans	139	0.576		7.035	6.755	0.280	0.0026
EQ4	b3f52	Bayley3	Builds bridge	977	1.440		7.035	7.070	− 0.035	
EQ5	b3g35	Bayley3	Raises self to standing position	390	− 13.766		− 6.455	− 6.381	− 0.074	0.0044*
EQ5	dg12	Denver	Pull to Stand	149	− 14.128		− 6.455	− 6.450	− 0.005	
EQ5	n29	DDI	Pulls up to standing position	3448	− 0.140		− 6.455	− 6.464	0.009	
EQ6	b3g42	Bayley3	Walks Series: Alone	516	− 9.466		− 2.580	− 2.575	− 0.005	0.0080*
EQ6	n42	DDI	Walks alone	3338	3.506		− 2.580	− 2.580	0.000	
EQ7	af33	ASQ-I	copies caregiver by drawing a circle	265	0.338		5.926	5.978	− 0.052	0.0007
EQ7	b3f43	Bayley3	Imitates Strokes Series: Circular	1719	0.167		5.926	5.918	0.008	
EQ8	b3g26	Bayley3	Sits without Support Series: 30 s	281	− 17.935		− 8.815	− 9.648	0.833	0.0000
EQ8	dg10	Denver	Sit No Support	87	− 18.213		− 8.815	− 9.834	1.019	
EQ8	n26	DDI	Sits in stable position without support	3528	− 2.184		− 8.815	− 8.756	− 0.059	

N = number of observed responses; ${\delta }_{i}$= Difficulty estimate of unconstraint item; ${\delta }_{q}$= Difficulty estimate for equate cluster; ${\eta }^{2}$ = effect size for item in DIF test; * P-value for ANOVA F-test < 0.05

## Discussion

The proliferation of many similar instruments, each defining an instrument-specific metric, produces measurements that are incomparable to other instruments. This paper presents a solution for the problem when strictly equivalent scales or items across different measurement instruments are lacking. Our method places similar items into equate clusters, determines which equate clusters to activate, and estimates difficulty parameters of items simultaneously for all instruments. All items within an active equate cluster receive the same difficulty estimates, thus providing a bridge between different instruments. The equate cluster method is intuitive for applied researchers and more flexible than current methodology.

The equate cluster method can be helpful in meta-analyses of individual person data from different studies that measure the same construct with different instruments. Assuming high-quality equate clusters, the method links data from different sources to the same scale. While our application is in child development, the same principles apply in other settings where combining data sets may offer new insights. Examples could include measuring quality of life, severity of disabilities, depression and physical activity. Also, when designing a new study, incorporating strategic overlap with other studies improves future equate cluster possibilities.

Equate clusters enhance existing methodologies for improving comparability. Current concurrent calibration methods place common-items into the same column [13, 19, 28, 29], thereby effectively constraining item parameters to be identical across studies. For example, the McHorney study developed a common metric for physical functioning using concurrent calibration, which requires that all studies are linked by identical common items [19]. Our method applies the constraints within the estimation algorithm. Since this does not require a specific organization of the data, we can easily equate multiple items within and across studies and within and across instruments. The method offers enough flexibility to deal with situations where common items are not perfectly identical or less abundant. Determining the optimal equate cluster composition and status (active or inactive) is a new type of modelling activity. Defining the optimal combination of equate clusters is a part of the modelling process that should not be taken lightly. Equate-group diagnostics like Fig. 5, may reveal that one or more items do not fit within the group. In such a case, we may need to remove a poorly fitting item from the equate cluster, split the equate cluster into two more homogeneous equate clusters, or decide to inactivate the equate cluster. There are no cut-and-dried criteria yet for such actions, but, as our simulations show, these decisions may have substantial effects on the solution. Based on our experience thus far, we make the following recommendations in working with equate clusters. First, collaborate with subject-matter experts to identify important similarities and differences in item formulations and a starting assignment of items into equate clusters. After formulating a probability model, assess the quality of equate clusters by studying the correspondence between the item characteristic curves and calculating equate fit statistics. Select equate clusters to activate, estimate ability, and, compare the ability distributions between studies, and evaluate whether any systematic differences are plausible. Try to distribute active equate clusters across the full range of the measurement scale. Finally, when the abilities of the samples are relatively uniform, try a model without any equate clusters, and see whether that solution may be preferable.

## Conclusions

In general, it is efficient to use and combine existing data sources to compare populations on a global health metric. However, more often than not, measurements made by different instruments are incomparable. We suggest the equate cluster method as an economical and exciting way to handle this problem, thus allowing for population-level comparisons on a global scale. We hope that the broader use of equate clusters may advance the utility of existing data for answering new questions.

## Supplementary Information

**Additional file 1:** R codes.

**Additional file 2:** Full tabulation of simulation results on the level of mis-alignment and correlation for the model with equate groups and the model without equate groups under the different simulation study conditions.

**Additional file 3:** Breakpoint of misspecification in logits where the model with equategroups still outperforms the model without equate groups.

## Data Availability

No datasets were generated or analysed during the current study.

## Abbreviations

ECD: Early Childhood Development
SI: International System of Units
GCDG: Global Child Development Group
NEAT: Non-equivalent anchor test
D-score: Generic score for child development
DDI: Dutch Development Instrument
BSID-III: Bayley Scales of Infant and Toddler Development, third edition
ASQ: Ages and Stages Questionnaire
Denver: Denver Developmental Screening test
DIF: Differential Item Functioning
