# Supplementary material for: Harmonizing measurements: establishing a common metric via shared items across instruments
Source: Popul Health Metr. 2024 Nov 7;22:30. doi: 10.1186/s12963-024-00351-z (PMC11546590; doi:10.1186/s12963-024-00351-z)
Supplement: Supplementary file 1 — Additional file 1: R codes. [file 12963_2024_351_MOESM1_ESM.pdf]

Appendix A: R codes

Pairwise Estimation of the Rasch Model under Item Equating

This function uses pairwise conditional likelihood estimation for estimating item parameters in the Rasch model.

Author

Alexander Robitzch (`sirt` author), Stef van Buuren

Arguments

data A matrix or a data frame. Item responses should be coded as `1` (pass) or `0` (fail). Missing responses are allowed and must be coded as `NA`.

equate Optional list with elements corresponding to a character vector of names of variable to be equated. The default (`NULL`) indicates that no equating takes place.‘

itemcluster Optional integer vector of itemcluster. Different integers correspond to different item clusters. The default (`NULL`) indicated that no item cluster is set.

b_fixed Numeric, named vector used for fixing item parameters, whose value are the parameters to be fixed. `names(b_fixed)` indicates the column in the data to which the fixed value applies.

b_init Numeric, named vector of initial item difficulty estimates. Under the default (`NULL`) values initial values are calculated internally.

zerosum Optional logical indicating whether item difficulties should be centered in each iteration. The default is that no centering is conducted.

count A table of counts `t(data == 0)` times `data == 1`.Items not present in `count` are silently discarded from the estimation process. The default (`NULL`) calculates the count table from the data. This will increase execution time substantially if `ncol(data)` is large, e.g. several hundreds of items.

conv Convergence criterion in maximal absolute parameter change

maxiter Maximal number of iterations

progress A logical which displays the iterative process. Default is `FALSE`.

compatible A logical indicating whether the function should return a list element called `X01`. For the Rasch model, this element is equivalent to element `X`, which is always returned. In order to save space, the default is set to `compatible = FALSE`. If you intend to use functions from the `eRm` package, set `compatible = TRUE`.

Value

Fitted object that extends class `c("dRm","Rm","eRm")` that is defined and understood by the `eRm` package. Note that the component `X01` is equivalent to `X` (the input data), and is not returned by default in order to save space. If you need any function from the `eRm` package that needs `X01`, specify the `compatible = TRUE` argument. The `rasch()` function adds several additional elements, most for compatibility with `sirt`.

equate The `equate` argument

b_fixed The `b_fixed` argument

itemcluster The `itemcluster` argument

b Vector of item difficulties‘

item Data frame of item parameters `N`, `p` and item difficulty

count The count table that is used by the algorithm. May differ from the count argument.

dropped Items that were dropped because they were not present in the count table (if specified)

Details

This function is based on `rasch.pairwise.itemcluster` from `sirt`, additional detail and examples can be found.

The `rasch()` function extend the original by incorporating the 'equate} argument. This facility allows the users to specify that Rasch parameters corresponding to different columns should receive identical values. In addition, some of the arguments have been changed to a more robust and friendly format (e.g. named vectors).

Formulas for asymptotic standard errors of this pairwise estimation method are described in Zwinderman (1995).

References

van der Linden, W. J., & Eggen, T. J. H. M. (1986). *An empirical Bayes approach to item banking.* Research Report 86-6, University of Twente.

Zwinderman, A. H. (1995). Pairwise parameter estimation in Rasch models. *Applied Psychological Measurement*, **19**, 369-375.


```
rasch <-
function (data, equate = NULL, itemcluster = NULL, b_fixed = NULL,
    b_init = NULL, zerosum = FALSE, count = NULL, conv = 1e-05,
    maxiter = 3000, progress = FALSE, compatible = FALSE, ...)
{
    call <- match.call()
    if (any(sapply(data, max, na.rm = TRUE) > 1))
        stop("Some items have scores > 1")
    if (any(sapply(data, min, na.rm = TRUE) < 0))
        stop("Some items have scores < 0")
    count_items <- colnames(count)
    data_items <- colnames(data)
    itemset <- count_items[count_items %in% data_items]
    Aij <- count[itemset, itemset]
    X01 <- data
    if (!is.null(itemset))
        data <- data[, itemset]
    dropped <- setdiff(names(X01), names(data))
    data <- as.matrix(data)
    p <- colMeans(data, na.rm = TRUE)
    N <- colSums(1 - is.na(data))
    I <- ncol(data)
    if (is.null(b_init))
        b_init <- -stats::qlogis(p)
    b <- b_init
    if (!is.null(b_fixed)) {
        b_fixed <- b_fixed[names(b_fixed) %in% names(b_init)]
        b[names(b_fixed)] <- b_fixed
```

```r
        exp_b_fixed <- exp(b_fixed)
        zerosum <- FALSE
    }
    if (is.null(count)) {
        data[is.na(data)] <- 9
        Aij <- t(data == 0) %*% (data == 1)
    }
    orphans <- NULL
    flags <- rowSums(Aij) == 0
    if (any(flags)) {
        orphans <- dimnames(Aij)[[1]][flags]
        cat("Orphans found: ", orphans, "\n")
    }
    clusters <- unique(itemcluster[itemcluster != 0])
    for (cc in clusters) {
        icc <- which(itemcluster == cc)
        Aij[icc, icc] <- 0
    }
    nij <- Aij + t(Aij)
    eps0 <- eps <- exp(b)
    max.change <- 10
    iter <- 1
    if (is.null(orphans)) {
        while (max.change > conv & iter <= maxiter) {
            b0 <- b
            eps0 <- eps
            m1 <- matrix(eps0, I, I, byrow = TRUE) + matrix(eps0,
                I, I)
            g1 <- rowSums(nij/m1)
            eps <- rowSums(Aij)/g1
            b <- log(eps)
            if (!is.null(b_fixed)) {
                eps[names(exp_b_fixed)] <- exp_b_fixed
            }
            if (length(equate) > 0) {
                for (i in seq_along(equate)) {
                  pos <- match(equate[[i]], names(eps))
                  if (anyNA(pos))
                    cat("\n Equate ", names(equate)[i],
                      "  Item not found: ", equate[[i]][is.na(pos)])
                  eps[pos] <- weighted.mean(x = eps[pos], w = N[pos])
                }
            }
            if (zerosum) {
                b1 <- -log(eps)
                b2 <- b1 - mean(b1)
                eps <- exp(-b2)
            }
            dif <- abs(b - b0)
            if (any(is.na(dif))) {
                pars <- names(b)[[is.na(dif)]]
                stop("Cannot estimate parameter: ", paste(pars))
            }
```

```
            max.change <- max(dif)
            if (progress) {
                cat("PL Iter.", iter, ": max. parm. change = ",
                  round(max.change, 6), "\n")
                flush.console()
            }
            iter <- iter + 1
        }
    }
    item <- data.frame(N = N, p = p, b = log(eps))
    if (is.null(itemcluster)) {
        itemcluster <- rep(0, I)
    }
    item$itemcluster <- itemcluster
    res <- list(X = as.data.frame(X01), count = Aij, dropped = dropped,
        orphans = orphans, model = "RM", equate = equate,
        itemcluster = itemcluster, b_fixed = b_fixed, zerosum = zerosum,
        loglik = 0, npar = I, iter = iter, convergence = conv,
        item = item, b = log(eps), etapar = -log(eps), se.eta = rep(NA,
            length(eps)), hessian = NULL, betapar = -log(eps),
        se.beta = rep(NA, length(eps)), W = diag(I), call = call)
    if (compatible)
        res$X01 <- res$X
    class(res) <- c("dRm", "Rm", "eRm")
    return(res)
}
```

R code for data simulation

Simulate the item level data

```
# input example
b <- list(seq(-3, 0, length = 10), seq(-1, 2, length = 10))
nsamp <- c(1000, 1000)
theta <- rnorm(n = sum(nsamp), sd = 2)


# define difficulty parameters as named vector
nit <- lapply(b, length)
index <- as.list(LETTERS[1:length(b)])
names(nit) <- index
names(b) <- index

itnames <- lapply(index, function(x) {
  if (is.null(names(b[[x]]))) {
    paste0(x, 1:nit[[x]])
  }
  else
    (names(b[[x]]))
})
names(itnames) <- index

# generate data
```

```r
n <- sum(nsamp)
dat <- sirt::sim.raschtype(theta, unlist(b))
colnames(dat) <- do.call(c, itnames)

head(dat, 10)
```

```
##    A1 A2 A3 A4 A5 A6 A7 A8 A9 A10 B1 B2 B3 B4 B5 B6 B7 B8 B9 B10
## 1   1  1  1  1  1  1  1  1  1   1  0  1  0  1  1  1  1  1  1   1
## 2   1  0  1  1  1  1  1  1  0   1  1  1  1  1  0  1  0  1  1   0
## 3   1  1  1  1  1  1  1  0  1   1  0  1  0  0  0  0  1  0  0   0
## 4   1  1  1  1  1  1  1  0  1   1  1  1  1  1  1  1  1  1  1   0
## 5   1  1  1  1  1  1  1  1  1   1  1  1  1  1  1  1  1  1  1   0
## 6   1  1  1  1  1  1  1  1  1   1  1  1  1  1  1  0  1  0  1   1
## 7   1  1  1  0  1  1  1  0  1   1  1  1  0  1  0  0  0  0  0   0
## 8   0  0  0  0  0  0  0  0  0   0  0  0  0  0  0  0  0  0  0   0
## 9   0  0  0  0  0  0  1  0  0   0  1  1  0  1  0  0  0  0  0   0
## 10  0  0  1  0  0  0  0  0  0   1  1  0  0  0  0  0  0  0  0   0
```

Fit the models on the data to compare methods

```r
# fit rasch model with items for all persons for true difficulties
fit <- dmetric::rasch(data = dat, equate=NULL)

#input equates
equates = list(c("A7","B1"), c("A9" ,"B3"), c("A10", "B4"))


# distribute data over samples
p <- c(0,cumsum(prop.table(nsamp))) #sampling probabilities
sample <- cut(1:nrow(dat), quantile(1:nrow(dat), prob = p, names = FALSE), include = TRUE)

samples <- split(dat, sample)
names(samples) <- index
samples_EQ1 <- lapply(index, function(x){
    samples[[x]][itnames[[x]]]
    })
samples_EQ <- do.call('bind_rows', samples_EQ1)

# fit Rasch model without equates
fit_noEQ <- dmetric::rasch(data = samples_EQ, equate=NULL)

# fit Rasch model with equate groups
fit_EQ <- dmetric::rasch(data = samples_EQ, equate=equates)
```
