# Supplementary material for: Harmonizing measurements: establishing a common metric via shared items across instruments
Source: Popul Health Metr. 2024 Nov 7;22:30. doi: 10.1186/s12963-024-00351-z (PMC11546590; doi:10.1186/s12963-024-00351-z)
Supplement: Supplementary file 2 — Additional file 2: Full tabulation of simulation results on the level of mis-alignment and correlation for the model with equate groups and the model without equate groups under the different simulation study conditions. [file 12963_2024_351_MOESM2_ESM.pdf]

Appendix B: Full tabulation of simulation results on the level of mis-alignment and correlation for the model with equate groups and the model without equate groups under the different simulation study conditions.

Difficulty ranges	n equates	location of equates	cohort abilities	mis-alignment(γ)		correlation(ρ)	
				Equate groups	No equate groups	Equate groups	No equate groups
[-5,-3][3,5]	1	central	same abilities	0.055	0.596	0.999	0.998
[-5,-3][3,5]	1	in one instrument	same abilities	0.262	0.651	0.999	0.999
[-5,-3][3,5]	1	spread	same abilities	0.051	0.773	0.999	0.998
[-5,-3][3,5]	1	extreme end	same abilities	1.429	0.623	0.992	0.998
[-5,-3][3,5]	2	central	same abilities	0.036	0.657	1.000	0.998
[-5,-3][3,5]	2	in one instrument	same abilities	-0.035	1.094	1.000	0.994
[-5,-3][3,5]	2	spread	same abilities	0.002	0.326	0.999	0.999
[-5,-3][3,5]	2	extreme end	same abilities	0.348	0.584	0.999	0.997
[-5,-3][3,5]	5	central	same abilities	0.057	0.438	0.999	0.998
[-5,-3][3,5]	5	in one instrument	same abilities	-0.065	1.139	1.000	0.991
[-5,-3][3,5]	5	spread	same abilities	-0.090	0.029	0.999	0.999
[-5,-3][3,5]	5	extreme end	same abilities	-0.116	0.590	0.998	0.994
[-3,-0.1][0.1,3]	1	central	same abilities	0.073	0.416	0.999	0.996
[-3,-0.1][0.1,3]	1	in one instrument	same abilities	-0.163	0.483	0.999	0.995
[-3,-0.1][0.1,3]	1	spread	same abilities	-0.080	0.442	0.999	0.996
[-3,-0.1][0.1,3]	1	extreme end	same abilities	0.115	0.495	0.999	0.995
[-3,-0.1][0.1,3]	2	central	same abilities	-0.011	0.425	0.999	0.995
[-3,-0.1][0.1,3]	2	in one instrument	same abilities	-0.021	0.456	1.000	0.995
[-3,-0.1][0.1,3]	2	spread	same abilities	0.057	0.284	0.999	0.998
[-3,-0.1][0.1,3]	2	extreme end	same abilities	-0.067	0.411	0.999	0.996
[-3,-0.1][0.1,3]	5	central	same abilities	-0.049	0.256	0.999	0.997
[-3,-0.1][0.1,3]	5	in one instrument	same abilities	-0.021	0.430	0.999	0.994
[-3,-0.1][0.1,3]	5	spread	same abilities	-0.050	0.205	0.999	0.998
[-3,-0.1][0.1,3]	5	extreme end	same abilities	0.095	0.426	0.999	0.996

[-2,1][-1,2]	1	central	same abilities	0.002	0.134	0.999	0.998
[-2,1][-1,2]	1	in one instrument	same abilities	-0.078	0.175	0.998	0.996
[-2,1][-1,2]	1	spread	same abilities	-0.094	0.167	0.998	0.996
[-2,1][-1,2]	1	extreme end	same abilities	0.343	0.125	0.990	0.998
[-2,1][-1,2]	2	central	same abilities	-0.158	0.141	0.996	0.996
[-2,1][-1,2]	2	in one instrument	same abilities	0.064	0.124	0.999	0.997
[-2,1][-1,2]	2	spread	same abilities	0.052	0.115	0.998	0.997
[-2,1][-1,2]	2	extreme end	same abilities	0.071	0.137	0.999	0.998
[-2,1][-1,2]	5	central	same abilities	0.031	0.110	0.999	0.998
[-2,1][-1,2]	5	in one instrument	same abilities	0.006	0.119	0.999	0.997
[-2,1][-1,2]	5	spread	same abilities	0.108	0.120	0.998	0.997
[-2,1][-1,2]	5	extreme end	same abilities	0.034	0.115	0.999	0.998
[-4,-1,1][-1,3][3.1,5]	1	central	same abilities	0.156	0.468	0.998	0.996
[-4,-1,1][-1,3][3.1,5]	1	central	same abilities	0.173	0.377	0.999	0.998
[-4,-1,1][-1,3][3.1,5]	1	central	same abilities	-0.082	0.353	0.999	0.998
[-4,-1,1][-1,3][3.1,5]	1	central	same abilities	0.110	0.389	0.999	0.997
[-4,-1,1][-1,3][3.1,5]	2	spread	same abilities	0.243	0.498	0.999	0.998
[-4,-1,1][-1,3][3.1,5]	2	spread	same abilities	-0.099	0.462	0.999	0.998
[-4,-1,1][-1,3][3.1,5]	2	spread	same abilities	-0.111	0.581	0.999	0.997
[-4,-1,1][-1,3][3.1,5]	2	spread	same abilities	-0.164	0.487	0.999	0.998
[-4,-1,1][-1,3][3.1,5]	5	spread	same abilities	-0.017	0.315	0.999	0.997
[-4,-1,1][-1,3][3.1,5]	5	spread	same abilities	0.179	0.290	0.998	0.997
[-4,-1,1][-1,3][3.1,5]	5	spread	same abilities	0.055	0.262	0.999	0.997
[-4,-1,1][-1,3][3.1,5]	5	spread	same abilities	0.066	0.303	0.999	0.997
[-5,-3][3,5]	1	central	different abilities	-0.084	2.455	1.000	0.979
[-5,-3][3,5]	1	in one instrument	different abilities	-0.014	2.641	1.000	0.968
[-5,-3][3,5]	1	spread	different abilities	-0.013	2.499	1.000	0.979
[-5,-3][3,5]	1	extreme end	different abilities	2.503	2.503	0.970	0.970

[-5,-3][3,5]	2	central	different abilities	0.174	2.329	0.999	0.971
[-5,-3][3,5]	2	in one instrument	different abilities	-0.060	2.653	1.000	0.956
[-5,-3][3,5]	2	spread	different abilities	-0.019	2.411	1.000	0.967
[-5,-3][3,5]	2	extreme end	different abilities	0.336	2.317	0.999	0.968
[-5,-3][3,5]	5	central	different abilities	0.048	2.185	1.000	0.955
[-5,-3][3,5]	5	in one instrument	different abilities	0.014	2.785	1.000	0.929
[-5,-3][3,5]	5	spread	different abilities	0.024	2.218	1.000	0.959
[-5,-3][3,5]	5	extreme end	different abilities	0.005	2.514	0.999	0.952
[-3,-0.1][0.1,3]	1	central	different abilities	-0.021	2.060	1.000	0.829
[-3,-0.1][0.1,3]	1	in one instrument	different abilities	0.030	2.133	1.000	0.822
[-3,-0.1][0.1,3]	1	spread	different abilities	-0.020	2.083	1.000	0.830
[-3,-0.1][0.1,3]	1	extreme end	different abilities	-0.115	2.201	1.000	0.848
[-3,-0.1][0.1,3]	2	central	different abilities	-0.008	2.038	0.999	0.808
[-3,-0.1][0.1,3]	2	in one instrument	different abilities	0.079	2.135	0.999	0.820
[-3,-0.1][0.1,3]	2	spread	different abilities	0.010	2.088	1.000	0.822
[-3,-0.1][0.1,3]	2	extreme end	different abilities	-0.121	2.139	0.999	0.874
[-3,-0.1][0.1,3]	5	central	different abilities	-0.063	1.938	0.999	0.797
[-3,-0.1][0.1,3]	5	in one instrument	different abilities	-0.006	2.201	0.999	0.788
[-3,-0.1][0.1,3]	5	spread	different abilities	0.006	1.930	1.000	0.857
[-3,-0.1][0.1,3]	5	extreme end	different abilities	0.234	2.113	0.998	0.887
[-2,1][-1,2]	1	central	different abilities	-0.197	1.776	0.996	0.625
[-2,1][-1,2]	1	in one instrument	different abilities	0.027	1.832	0.999	0.650
[-2,1][-1,2]	1	spread	different abilities	0.021	1.803	0.998	0.612
[-2,1][-1,2]	1	extreme end	different abilities	-0.167	1.844	0.996	0.734
[-2,1][-1,2]	2	central	different abilities	0.080	1.826	0.998	0.620
[-2,1][-1,2]	2	in one instrument	different abilities	0.133	1.799	0.997	0.639
[-2,1][-1,2]	2	spread	different abilities	-0.085	1.795	0.998	0.680
[-2,1][-1,2]	2	extreme end	different abilities	0.022	1.835	0.999	0.796

[-2,1][-1,2]	5	central	different abilities	0.015	1.780	0.999	0.705
[-2,1][-1,2]	5	in one instrument	different abilities	0.060	1.768	0.999	0.657
[-2,1][-1,2]	5	spread	different abilities	-0.044	1.774	1.000	0.741
[-2,1][-1,2]	5	extreme end	different abilities	0.040	1.858	0.998	0.827
[-4,-1,1][-1,3][3.1,5]	1	central	different abilities	0.208	2.114	0.999	0.793
[-4,-1,1][-1,3][3.1,5]	1	central	different abilities	-0.066	2.184	0.999	0.783
[-4,-1,1][-1,3][3.1,5]	1	central	different abilities	-0.037	2.132	1.000	0.795
[-4,-1,1][-1,3][3.1,5]	1	central	different abilities	0.065	2.130	1.000	0.780
[-4,-1,1][-1,3][3.1,5]	2	spread	different abilities	0.078	2.227	1.000	0.826
[-4,-1,1][-1,3][3.1,5]	2	spread	different abilities	0.020	2.250	1.000	0.830
[-4,-1,1][-1,3][3.1,5]	2	spread	different abilities	-0.295	2.203	0.999	0.831
[-4,-1,1][-1,3][3.1,5]	2	spread	different abilities	0.084	2.259	1.000	0.829
[-4,-1,1][-1,3][3.1,5]	5	spread	different abilities	0.021	2.099	1.000	0.721
[-4,-1,1][-1,3][3.1,5]	5	spread	different abilities	0.006	2.087	1.000	0.715
[-4,-1,1][-1,3][3.1,5]	5	spread	different abilities	0.075	2.028	1.000	0.728
[-4,-1,1][-1,3][3.1,5]	5	spread	different abilities	0.008	2.120	1.000	0.721
