# Supplementary material for: Harmonizing measurements: establishing a common metric via shared items across instruments
Source: Popul Health Metr. 2024 Nov 7;22:30. doi: 10.1186/s12963-024-00351-z (PMC11546590; doi:10.1186/s12963-024-00351-z)
Supplement: Supplementary file 3 — Additional file 3: Breakpoint of misspecification in logits where the model with equategroups still outperforms the model without equate groups. [file 12963_2024_351_MOESM3_ESM.pdf]

Appendix C: Breakpoint of misspecification in logits where the model with equategroups still outperforms the model without equate groups

difficulty	number of equates	location of equates	theta	Correlation with Equate groups > Correlation No Equate groups	Mis-alignment with Equate groups > Mis-alignment No Equate groups
				Deviation in Logits	Deviation in Logits
[-3,-0.1][0.1,3]	1	extreme end	different	1.7	1.7
[-3,-0.1][0.1,3]	2	extreme end	different	2.0	2.0
[-3,-0.1][0.1,3]	5	extreme end	different	2.0	2.0
[-5,-3][3,5]	1	extreme end	different	2.0	2.0
[-5,-3][3,5]	2	extreme end	different	2.0	2.0
[-5,-3][3,5]	5	extreme end	different	2.0	2.0
[-2,1][-1,2]	1	extreme end	different	1.9	1.9
[-2,1][-1,2]	2	extreme end	different	2.0	2.0
[-2,1][-1,2]	5	extreme end	different	2.0	2.0
[-3,-0.1][0.1,3]	1	in one instrument	different	2.0	2.0
[-3,-0.1][0.1,3]	2	in one instrument	different	2.0	2.0
[-3,-0.1][0.1,3]	5	in one instrument	different	2.0	2.0
[-5,-3][3,5]	1	in one instrument	different	2.0	2.0
[-5,-3][3,5]	2	in one instrument	different	2.0	2.0
[-5,-3][3,5]	5	in one instrument	different	2.0	2.0
[-2,1][-1,2]	1	in one instrument	different	1.9	1.9
[-2,1][-1,2]	2	in one instrument	different	2.0	2.0
[-2,1][-1,2]	5	in one instrument	different	2.0	2.0
[-3,-0.1][0.1,3]	1	central	different	2.0	2.0
[-3,-0.1][0.1,3]	2	central	different	2.0	2.0
[-3,-0.1][0.1,3]	5	central	different	2.0	2.0
[-5,-3][3,5]	1	central	different	2.0	2.0
[-5,-3][3,5]	2	central	different	2.0	2.0
[-5,-3][3,5]	5	central	different	2.0	2.0
[-2,1][-1,2]	1	central	different	1.9	1.9
[-2,1][-1,2]	2	central	different	2.0	2.0
[-2,1][-1,2]	5	central	different	2.0	2.0
[-3,-0.1][0.1,3]	1	spread	different	2.0	2.0
[-3,-0.1][0.1,3]	2	spread	different	2.0	2.0
[-3,-0.1][0.1,3]	5	spread	different	2.0	2.0
[-5,-3][3,5]	1	spread	different	2.0	2.0
[-5,-3][3,5]	2	spread	different	2.0	2.0
[-5,-3][3,5]	5	spread	different	2.0	2.0
[-2,1][-1,2]	1	spread	different	1.9	1.9
[-2,1][-1,2]	2	spread	different	2.0	2.0
[-2,1][-1,2]	5	spread	different	2.0	2.0
[-3,-0.1][0.1,3]	1	extreme end	same	0.5	0.5
[-3,-0.1][0.1,3]	2	extreme end	same	0.5	0.5
[-3,-0.1][0.1,3]	5	extreme end	same	1.0	1.0
[-5,-3][3,5]	1	extreme end	same	0.7	0.7
[-5,-3][3,5]	2	extreme end	same	0.1	0.1
[-5,-3][3,5]	5	extreme end	same	1.2	2.0
[-2,1][-1,2]	1	extreme end	same	0.3	0.3
[-2,1][-1,2]	2	extreme end	same	0.4	0.4
[-2,1][-1,2]	5	extreme end	same	0.2	0.2
[-3,-0.1][0.1,3]	1	in one instrument	same	0.4	0.4
[-3,-0.1][0.1,3]	2	in one instrument	same	0.8	0.9
[-3,-0.1][0.1,3]	5	in one instrument	same	1.2	2.0
[-5,-3][3,5]	1	in one instrument	same	0.8	0.8
[-5,-3][3,5]	2	in one instrument	same	1.0	1.4
[-5,-3][3,5]	5	in one instrument	same	1.7	2.0
[-2,1][-1,2]	1	in one instrument	same	0.0	0.0
[-2,1][-1,2]	2	in one instrument	same	0.6	0.6
[-2,1][-1,2]	5	in one instrument	same	0.5	0.5
[-3,-0.1][0.1,3]	1	central	same	0.6	0.6
[-3,-0.1][0.1,3]	2	central	same	0.6	0.7
[-3,-0.1][0.1,3]	5	central	same	0.7	1.5
[-5,-3][3,5]	1	central	same	0.6	0.6
[-5,-3][3,5]	2	central	same	0.7	1.1
[-5,-3][3,5]	5	central	same	0.8	1.5
[-2,1][-1,2]	1	central	same	0.1	0.1
[-2,1][-1,2]	2	central	same	0.3	0.3
[-2,1][-1,2]	5	central	same	0.4	0.6
[-3,-0.1][0.1,3]	1	spread	same	0.4	0.4
[-3,-0.1][0.1,3]	2	spread	same	0.6	0.6
[-3,-0.1][0.1,3]	5	spread	same	0.9	1.2
[-5,-3][3,5]	1	spread	same	0.9	0.9

[-5,-3][3,5]	2	spread	same	0.2	0.2
[-5,-3][3,5]	5	spread	same	0.5	0.1
[-2,1][-1,2]	1	spread	same	0.1	0.1
[-2,1][-1,2]	2	spread	same	0.1	0.1
[-2,1][-1,2]	5	spread	same	0.3	0.3
